# Evaluation of the Influence of Bolt Fastener Spacing on the Elastic Critical Load from the Lateral-Torsional Buckling Condition of Built-Up Bending Members

**DOI:** 10.3390/ma17143392

**Published:** 2024-07-09

**Authors:** Rafał Piotrowski, Andrzej Szychowski

**Affiliations:** Faculty of Civil Engineering and Architecture, Kielce University of Technology, 25-314 Kielce, Poland; aszychow@tu.kielce.pl

**Keywords:** built-up 2C section, transverse bending, elastic critical load, the lateral-torsional buckling, experimental studies

## Abstract

In an experimental study of two-branched beams bent transversely about the major stiffness axis, the elastic critical load from the lateral-torsional buckling condition was determined. The tests were conducted on simply supported two-branch beam models with a built-up section consisting of two cold-formed channel members (2C) bolted back-to-back. The bolts were located at the mid-height of the built-up cross-section. Five groups of members differing in longitudinal bolt spacing were examined. The models were gravitationally loaded (using ballast) at the centre of the beam span. This approach eliminated the undesirable effect of the lateral support of the beam, e.g., by the actuator head. The critical load, measured by the concentrated transverse force (*P_z_*_,*cr*_), was determined using the modified Southwell method. It has been experimentally shown that, in built-up beams, there is an influence of bolt spacing on the elastic critical load from the lateral-torsional buckling condition. The lowest critical load capacity and the most non-linear behaviour of the built-up member were observed in beams bolted with only three bolts (at the supports and in the middle of the span). However, the experimental results obtained in this study show that increasing the number of bolts above a certain level (in the case of the tested models, it was seven bolts) does not result in a further increase in the critical load, which is a surprising result. The obtained values were 15 to 23% lower than the critical load determined numerically by the finite element method (*LTBeamN*) for an analogous element with a uniform I-section.

## 1. Introduction

Cold-formed, thin-walled, open cross-section members are commonly used in modern metal construction. Their fundamental advantage is the low weight of the members and structures made of them [1]. This is due to the small wall thickness of the section profiled in this way. Consequently, cold-formed members are sensitive to various forms of global instability (buckling, lateral-torsional buckling) as well as local and distortion buckling [1,2].

The theoretical and experimental research conducted over the last four decades has allowed for a relatively accurate understanding of the instability phenomena occurring in single thin-walled members. In this case, the research methods and the well-developed computational models are well known [3,4,5]. Among other things, the behaviour under the load of single thin-walled members used in metal construction is well known—for instance, in purlins, wall girders or lightweight structures with smaller spans [1,3].

However, the further development of constructions made of cold-formed profiles requires achieving higher resistances of members (in the case of compression and bending) while taking into account their sensitivity to various modes of local and distortion buckling and spatial forms of global stability loss (flexural buckling, flexural-torsional buckling, and lateral-torsional buckling). In the case of thin-walled open sections, the sensitivity to flexural-torsional buckling and lateral-torsional buckling is caused by low torsional stiffness.

To increase the load-bearing capacity of cold-formed steel members, built-up members bolted back-to-back [1] are used in certain types of construction (e.g., portal frames, frame structures, or beam grillages). In this case, the additional difficulty in creating engineering computational models is the complex interaction phenomena: member branch–sheared bolts–member branch.

In the European standards [3,6] for the design of steel structures, no information is provided on determining the elastic critical load from the lateral-torsional buckling condition for built-up members bending about the material axis (i.e., the axis intersecting the material of both branches). This relates to the beams whose members are back-to-back bolted so that the material axis is also the axis of the major stiffness of the built-up cross-section. The currently applicable European standard—Eurocode 3-1-1 [6]—only provides guidelines for the maximum spacing of the bolt connectors *l*_1_ = 15*i_min_* (where *i_min_*—minimum radius of gyration of the cross-section of a single branch) for built-up members loaded in axial compression. Only when the above condition is met can such members be treated as uniform with a compact cross-section. It should be noted that it is often uneconomical to comply with the above condition due to the need for many bolts and the dense hole-punching of members. Due to the fact that the above-mentioned bolt spacing applies only to axially compressed columns, the question arises whether it can be applied to bending beams that are in a different load and stress state. The form of the spatial loss of stability of a bending member is also more complex than that of an axially compressed member. Both bending and torsional deformations characterise it.

The lateral-torsional buckling phenomenon is well recognised for bi-symmetrical single sections (e.g., hot-rolled or welded I-beams). In this area, there is a whole collection of work on experimental research, theoretical and analytical research, and numerical simulations. For example, a compact list of articles describing problems that have already been solved can be found in the works [7,8,9].

Extensive theoretical and analytical considerations on the behaviour of built-up members connected discretely by bolts are provided in the work [10]. The research was carried out on members not susceptible to local buckling. Beams bending against the axis of minor stiffness of the two-branch cross-section, torsion-loaded members, and compression columns are included. For up to seven connections distributed along the length of the member, closed-form analytical solutions were obtained. Among other things, the influence of the number of bolt connectors on the critical force of flexural buckling was considered. However, the phenomenon of lateral-torsional buckling was not analysed.

Most of the works related to the behaviour and load-bearing capacity of built-up members considered, among others, (a) experimental and numerical investigations of axially compressed columns with a cross-section composed of two cold-formed steel channels, e.g., [11,12], (b) compressed members with a cross-section composed of two Sigma sections e.g., [13], (c) interactions of the forms of buckling (local, distortion, and lateral-torsional) of transversely bent two-branch cross-section beams in stainless steel [14,15], (d) experimental studies of members composed of three or four channel sections [16], (e) the analysis of members of a 2C cross-section with an additional plate stiffening the compression flange [17], (f) the optimisation of transversely bent beams with double Sigma cross-sections [18], (g) experimental, numerical, and analytical investigations of the bending stiffness of a 2C member with respect to the minor axis of the built-up cross-section [19], (h) the design and analysis of the resistance of bipolarly pre-stressed, closely spaced, and built-up compression members constructed from a pair of channels [20,21], (i) the behaviour of built-up members connected by flanges, e.g., [22,23], (j) numerical simulations of the stability of double Sigma sections containing clearances between members [24].

Experimental investigations into the stability and ultimate capacity of compression built-up columns are presented in the papers [11,12]. The members consisted of back-to-back cold-formed steel (CFS) channel bars joined by self-drilling screws. The experiments carried out made it possible to quantify, among other things, the effect of the longitudinal screw arrangement and the support conditions at the support nodes on the performance of the built-up member. The following interactions were observed: local–global and distortion–global forms of stability loss of the tested models. Based on the experiments, the stiffness of the elastic restraint of the members at the support nodes was estimated. For this purpose, the classical Southwell method was used [25] to determine the elastic critical load from the load–lateral displacement relationship of the member. The results of the research have made it possible to extend the application of the Direct Strength Method (DSM) [1,5] to the design of built-up members.

The works [14,15] present experimental tests and numerical simulations of transversely bent stainless steel two-branch beams. The beam cross-sections have shown sensitivity to local buckling. In the four-point bending test of unbraced beams, the interaction phenomenon of local and lateral-torsional buckling was observed. An original load generation system was used to reduce the effect of actuator heads laterally supporting the model. Unique four-joint frames were used to transfer the load from the actuators in the form of concentrated forces applied to the shear centre of the built-up cross-section. However, in the opinion of the authors of this paper, even such a complex method of loading with actuators does not guarantee that only gravity loading can be achieved without introducing an undesirable—in this case, a lateral elastic support effect on the model. This effect affects the lateral-torsional buckling phenomenon under investigation and depends on the elastic support index in the lateral direction, which is difficult to determine.

In the literature on the subject, there are no works describing the phenomenon of the lateral-torsional buckling of built-up beams, in which the influence of the longitudinal bolt spacing only on the critical moment of lateral-torsional buckling was investigated. There is also a lack of experimental studies on the lateral-torsional buckling of beams with built-up cross-sections where a gravity load was applied (without inducing a lateral support effect). Gravity loads (using applied ballast) have so far been used only in tests of single elements, as exemplified by [26].

Such studies of built-up cross-sections beams would allow for the analysis of only the lateral-torsional buckling phenomenon, without the influence of interactions with other instability phenomena, such as local and/or distortion buckling. This decoupling of the phenomena that precede the exhaustion of the member’s load-bearing capacity would facilitate the development of engineering computational models. It could provide a starting point for further, more advanced analyses. The elastic critical moment (*M_cr_*) is an important design parameter. From this, the so-called relative slenderness (${\bar{\lambda }}_{LT}$) and the reduction factor for lateral-torsional buckling ($\chi_{LT}$) are determined, which ultimately allows for assessing the safety of the steel bending member.

In order to experimentally determine the elastic critical load of the steel bar (and, consequently, the critical moment *M_cr_*), an appropriate method of analysing the experimental data must be used.

For the experimental determination of the elastic critical load of compression members, e.g., [12,27,28,29] or bending beams [29,30], the Southwell method is used [25]. Classically, the method has been used to experimentally determine the critical force of axially compressed columns possessing geometric imperfections with a near buckling form. In this case, even at relatively low loads, the form of displacement increments achieves a form close to buckling according to the first (smallest) critical load. Based on the experimentally determined load–deflection (*P*–*ν*) static equilibrium path, a “Southwell plot” is drawn up, and the elastic critical load is determined. This approach has already been successfully verified experimentally. However, some researchers obtained results with significant errors resulting from the different form of geometric imperfections of the test models from the buckling form. The paper [28] numerically investigates and identifies the reasons for possible inconsistencies between the experimental results of the critical force of flexural buckling determined by the classical Southwell method and theoretical calculations.

The Southwell method can also be used to test the instability of plates [29,31] and the local buckling of thin-walled Class 4 members composed of plates, e.g., [32]. In this case, there may be even greater inconsistencies (especially for complex stress states) due to potential differences between the random form of the local imperfections of the walls of the thin-walled section and the form of the buckling for the first eigenvalue.

In the paper [32], in which the so-called local critical bi-moment (*B_cr_*) was determined on the basis of experimental tests on warping torsion thin-walled members with Class 4 cross-sections (according to [6]), it was observed that the different shape of geometrical imperfections (in relation to the expected form of local buckling) can significantly delay the occurrence of deflections that can be used to construct “Southwell plots”. The analysis of such experimental situations has led to the observation of the relevance of the phenomenon of so-called “deflections ordering” [31,32].

This type of behaviour of a structural member, namely, the delayed occurrence of displacements consistent with the first form of the loss of stability, may also occur when experimentally determining the elastic critical load of transversely bent beams with a 2C built-up cross-section. This phenomenon is discussed in detail in Section 4.1.

Due to the knowledge gap presented above, this research was aimed at the experimental verification of the following hypotheses: (1) in transversely bending built-up beams, there is an eigenvalue of the elastic critical load, (2) the critical load depends on the longitudinal spacing of the bolted connectors, (3) loading the test model with ballast may allow only the gravity load to be obtained without the disadvantages (in this case, the influence of lateral support), (4) there is a spacing of fasteners that is more significant than in the case of compression members and economically justifiable, which provides an equivalent critical load resistance in relation to a member with an equivalent uniform cross-section.

The main objective of the experimental study was to assess the influence of the longitudinal spacing of the bolt connectors on the elastic critical load capacity from the lateral-torsional buckling condition. The members analysed had a bi-symmetrical cross-section, a class no higher than 3, and, following [6], were composed of two channels (2 × C) placed back-to-back and fastened with bolts. A concentrated force load applied at the level of the upper flange at the centre of the span of the beam has been considered.

## 2. Experimental Studies

### 2.1. Details of Test Models

An experimental assessment of the influence of longitudinal bolt spacing on the elastic critical lateral-torsional buckling resistance of a built-up member was carried out for beams with a bi-symmetrical cross-section composed of two cold-formed channels with geometric proportions of, at most, Class 3 (i.e., insensitive to local buckling in the elastic range). The plain channel type adopted (with no free flange edge bends) eliminated the possibility of distortion buckling.

**Figure 1:**
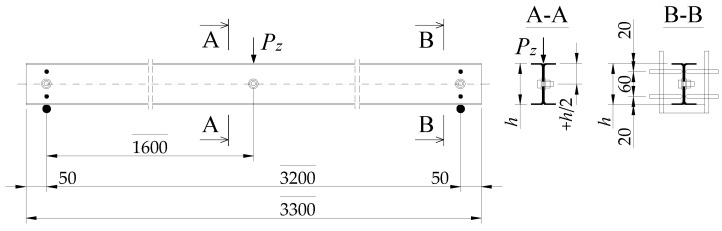
Support and loading diagram of the test model.

The static scheme chosen for the experimental study was that of a fork-supported 2C built-up member, where the number of bolts varied from 3 to 11 along its length. The test model was loaded with a concentrated transverse force *P_z_* applied to the top flange of the built-up cross-section at the midspan of the beam, according to the scheme shown in Figure 1. The loading was carried out by the force of gravity, using weights (ballast) placed on a platform suspended under the model.

The tested models’ theoretical span in the support bearings was *L* = 3.2 m. The bolt fasteners were located at the mid-height of cross-section 2C and distributed evenly along the length of the element in five variations, from 3 to 11 bolts, with an increase of 2 bolts in successive variations (W1 to W5). It was assumed that the smallest bolt spacing, for variant W5, would be approximately 2.5 × 15*i_min_* (*i_min_*—minimum radius of gyration of a single channel bar). Note: the value of 15*i_min_* is derived from the requirement of Eurocode 3-1-1 [6] for the spacing of fasteners for a compressed built-up member treated as a uniform section in the calculations. In the case of the members in the study, compliance with this condition would be a completely uneconomic solution. Along the length of the model, 27 bolts would have to be used. They should be spaced about 12.3 cm apart.

A total number of 22 built-up models (2C) were prepared for experimental testing, 4 for each of the 5 variants (W1 to W5) of longitudinal bolt placement and 2 built-up models (2C) for testing the support settings, sensors placement, load application method, etc. This number of test models allowed for the statistical evaluation of (1) the geometrical sizes of the component bars, (2) the bolt hole spacing and diameters, (3) the measurements of displacements and angles of rotation as a function of the applied load, and (4) the experimental critical load capacity from the lateral-torsional buckling condition for individual variants.

Table 1 shows the designations of the different variants, the number of bolts used, and their spacing.

**Figure 2:**
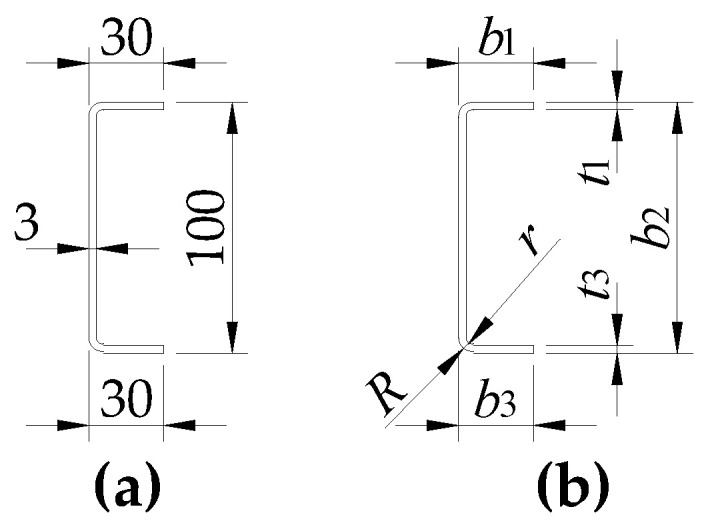
Cross-section of a single branch: (**a**) nominal dimensions; (**b**) measured dimensions.

A thermomechanically rolled sheet with a nominal thickness of 3 mm and S355MC grade steel [33] was used for constructing the models. Strips approximately 150 mm wide and 3300 mm long, separated by 70 mm wide strips, were cut parallel to the rolling direction from coils of 2000 mm wide sheet steel, from which samples were taken to test the material’s mechanical properties. In strips of approx. 150 mm in width (the width of the strip needed to make a single C section was determined according to [34]), the position of the bolt holes was traced. The holes were punched out according to the adopted spacing for variants W1 to W5 (cf. Table 1). After punching holes, they were carefully bent (with an inner bending radius of about 3 mm) using an automatic bender into C-sections marked with symbols from w1-*i* to w5-*i* (where *i* = 1, 2, 3, …, 8). The theoretical nominal dimensions (following the contour of the section) of the C100 × 30 × 3 channels used and the designations of the dimensions measured on each C-section are shown in Figure 2.

The dimensions of the models (*b*_1_, *b*_2_, *b*_3_, *t*_1_, *t*_3_) along the outer contour of the cross-section of a single C-section (Figure 2b) were determined in at least five sections along the length of the model. In each variant (from w1-*i* to w5-*i*), the following were measured for two models: (a) external (*R*) and internal (*r*) bend radii (Figure 2b), (b) bolt hole diameters, (c) longitudinal hole spacing, and (d) the location of holes at the height of the C-section. On this basis, a statistical evaluation of the above-mentioned geometric quantities of the members was carried out, from which it can be seen that the C-models were made with great care (the coefficients of variation of the parameters mentioned above in the vast majority of cases were below 1.0%). The mean values, standard deviations, and coefficients of variation of the measured dimensions for the w4-4 model are contained in Table 2.

The inventoried models of the single C-section, marked by the symbols w1-*i* to w5-*i* (where *i* = 1, 2, 3, …, 8), were assembled into target 2C test models and bolted together with M12, class 8.8 bolts. The bolts were tightened with a torque spanner to 0.5 torque for full compression [35,36]. The 2C research models are labelled according to the variants adopted (cf. Table 1), with symbols W1-*j* to W5-*j*, where *j* = A, B, C, and D denote the next four models of the given variant (group). For example, four 2C test models were built for variants W1: W1-A, W1-B, W1-C, and W1-D.

The measurement of the summed flange widths of the 2C built-up models (in three cross-sections, including the centre of the span) showed high accuracy in the assembly of the C-section into the 2C test pair (differences did not exceed 0.5%). The test programme was designed to experimentally determine the elastic critical load from the lateral-torsional buckling condition of a fork-supported 2C built-up member under only gravity loading. The geometric proportions used and the plain C-section type eliminated the possibility of local or distortional buckling in the elastic range.

### 2.2. Examination of Material Properties

The properties of the steel (basic material) were tested on samples cut in the rolling direction from 70 mm wide strips taken from the sheet metal intended for test models. Eight specimens, designated P-*k* (where *k* = 1, …, 8), were prepared for testing.

**Figure 3:**
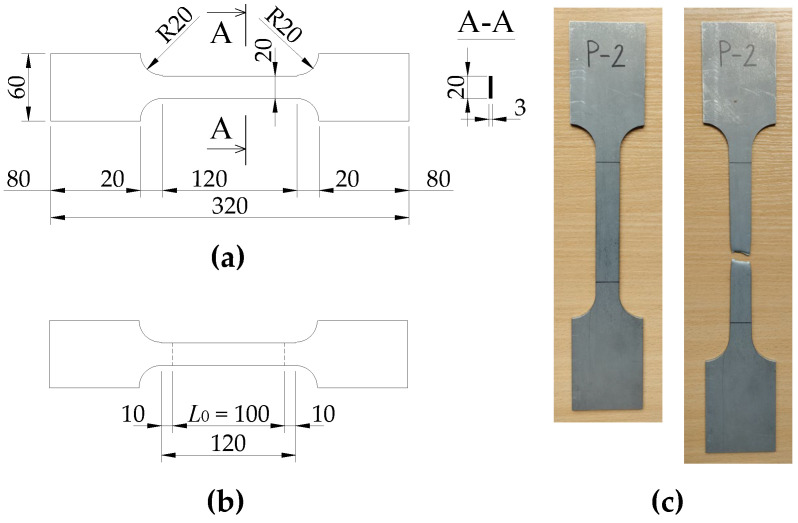
(**a**) Nominal dimensions of the test specimen for the test of the mechanical properties of the material; (**b**) Determination of the initial measuring length *L*_0_; (**c**) Test specimen P-2 before and after testing.

According to Figure 3a, samples with geometries were made in accordance with the Standard [37] as non-proportional. A Zwick/Roell Z250 testing machine was used for the tests.

The Young’s modulus, upper and lower yield strengths (*R_eH_* and *R_eL_*), and tensile strength (*R_m_*) were determined experimentally for specimens with a labelled initial measurement length *L*_0_ (according to Figure 3b). The mean values, standard deviations, and coefficients of variation of the mechanical properties of the steel (basic material) are summarised in Table 3.

The results obtained for the upper and lower yield strength and tensile strength are within the range of variation for S355MC steel according to the metallurgical standard [33].

### 2.3. Test Bench and Measuring Equipment

**Figure 4:**
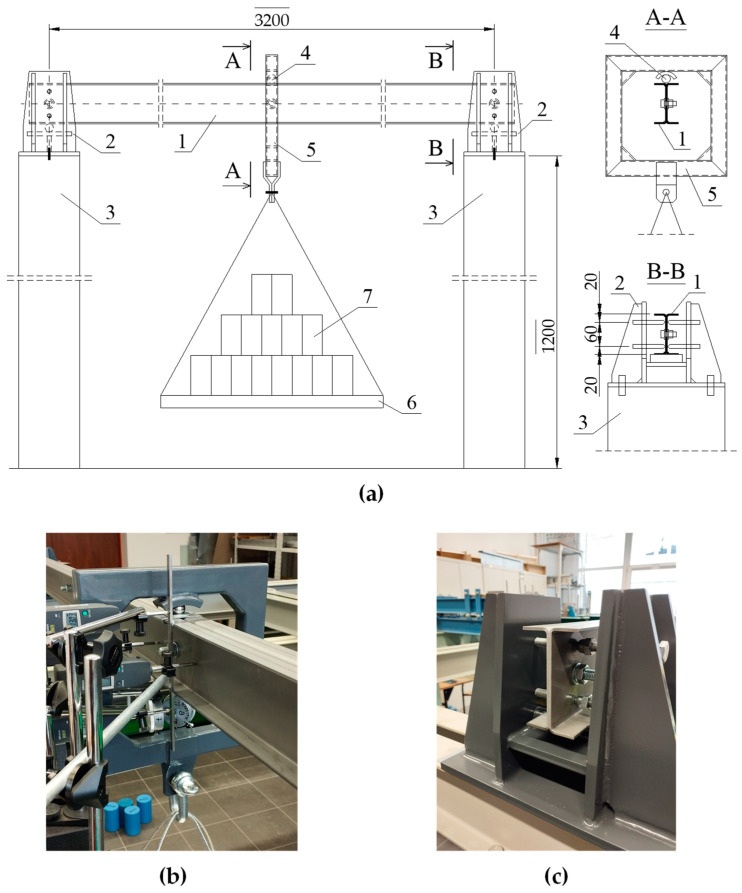
(**a**) Test bench diagram: 1—test model 2C, 2—support bearing, 3—support structure, 4—steel ball, 5—sling frame, 6—load platform, 7—weights; (**b**) Photograph of the sling frame; (**c**) Photograph of the support bearing.

A schematic diagram of the test stand for determining the transverse critical force *P_z_*_,*cr*_ from the lateral-torsional buckling condition of a 2C built-up beam is shown in Figure 4.

The tests were carried out on a bench with adequate model capture at the support nodes (Figure 4a,c). Weights were applied to the loading platform to create a load in the form of a concentrated force applied at the centre of the model span. The platform was suspended from a dedicated sling frame (Figure 4b), which transmitted force via a steel ball to the top flange of the 2C built-up cross-section. The ball sat in the hole of the steel bracket surrounding the top flanges at the point of load application, eliminating any deformation of the 2C built-up cross-section under concentrated force. This gravitational loading method eliminated the undesirable effect of the lateral support of the test beam at the point of load application, e.g., by the actuator head. This was due to the predicted form of the lateral-torsional buckling according to a single half-wave sine function with maximum displacements in the centre of the model span. This approach was also necessary because of the required accuracy of measurements of geometric values (e.g., lateral displacement and torsion angle) as a load function. Measurements of horizontal and vertical displacements at the centre of the model span and longitudinal horizontal displacements at the support node (measurement of cross-section warping at the support) were made using electronic and dial (mechanical) displacement sensors with a measurement accuracy of 0.01 mm (electronic and dial sensors) and 0.001 mm (precision dial sensors—warping measurement). The results of the measurements were used to experimentally determine the elastic critical load using a modification of the Southwell method.

To achieve the intended boundary conditions for the beam’s support, which prevented rotation about the longitudinal axis while allowing for warping of the cross-sections on the supports, four-point forked brackets (see Figure 4a,c) were used, arranged in pairs at the height of the webs of the 2C built-up cross-section.

**Figure 5:**
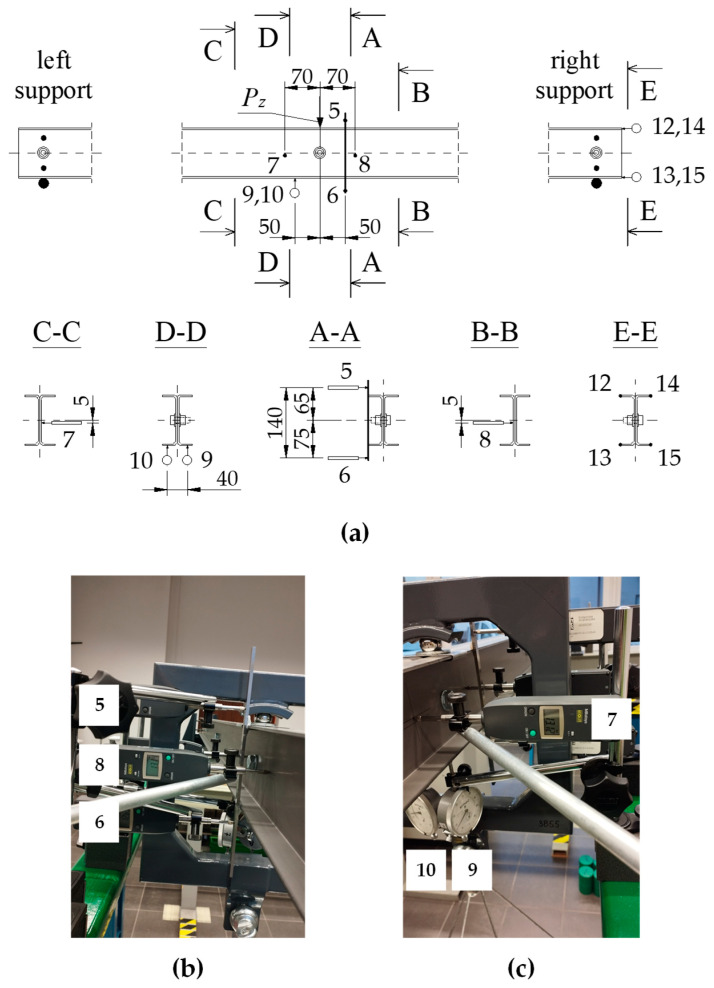
(**a**) Schematic of displacement sensor locations; (**b**) Sensors 5, 8, and 6; (**c**) Sensors 7, 9, and 10.

Figure 5a shows a schematic and labelled arrangement of the displacement sensors: 5, 6, 7, and 8—electronic sensors (24/0.01 mm), 9 and 10—dial indicators (10/0.01 mm), and 12, 13, 14, and 15—precision dial indicators (1/0.001 mm). Figure 5b,c show a view of the displacement sensors during the W3-C model test.

For the beam static scheme used in the experiment (Figure 1), the extreme value of displacements (horizontal deflections and torsion angle) associated with the first eigenvalue of the elastic critical load occurred in the middle of the span. At the same time, a particular sling frame (see Figure 4a,b) was used to generate a concentrated load. The technically sound way of imposing a gravitational load prevented the sensors from being positioned precisely in the middle of the span of test model 2C. Sensors 5, 6, 7, 8, 9, and 10 were located accordingly, in sections slightly away from the centre of the model span (up to 2.5% of the design span) (see Figure 5a). In this case, the difference in displacement readings did not exceed 0.3%.

During the experimental tests, the following were measured (cf. Figure 5a): (1) the vertical deflection of the beam in cross-section D-D (sensors 9 and 10), (2) the rotation angle of the 2C-section in cross-section A-A (sensors 5 and 6), (3) the horizontal displacements in cross-sections B-B (sensor 8) and C-C (sensor 7), (4) the warping of the built-up cross-section 2C (i.e., the translation of the free edges of the channel flanges along the longitudinal axis of the member) in the correct support bearing (sensors 12, 13, 14, and 15).

**Figure 6:**
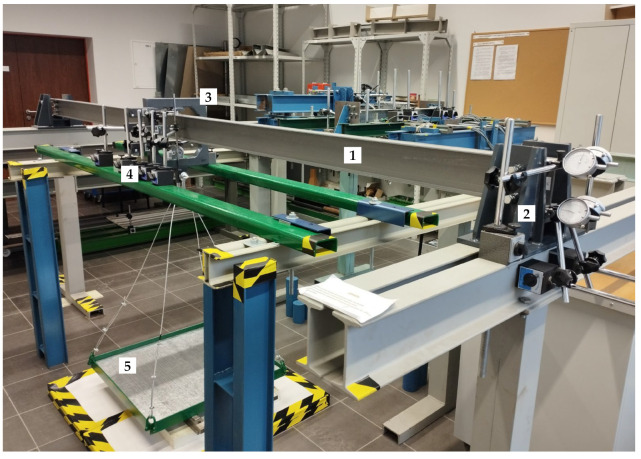
Model W1-A prepared for experimental testing: 1—test model, 2—support bearing, 3—sling frame, 4—sensors mid-span of the beam, and 5—load platform.

A general view of the W1-A (third bolts) bench prepared for experimental testing is shown in Figure 6.

### 2.4. Measurement of Geometric Imperfections

The experimental investigations were preceded by an assessment of the geometric imperfections of the single C channels and an approximate measurement of the lateral pre-bending of the built-up 2C cross-section member.

In most cases, the individual C-members showed lateral pre-bending as a single half-wave with maximum inaccuracy (deflection towards the edges of the outer flanges) near the centre of the length. After the individual C-members have been assembled back-to-back to form the 2C-member and before they are bolted together, a gap of 0.5 to max. 2.0 mm for most test pairs was observed. After connecting the C-sections into the built-up target model 2C, mutual straightening of the members was observed, corresponding to the number of connectors used. Inaccuracies in the web fit that occurred in some cases were measured with feeler gauges. The W1 (3 bolts) and W2 (5 bolts) models had the maximum gaps between the connected back-to-back bars. In most cases, the gaps between the 2C model branches did not exceed 0.3 mm (max. 0.4 mm for W1) and were located near the centre of the respective bolt spacing.

As each group (W1 to W5) provided for a bolt in the centre of the beam span (see Table 1), where the maximum flexural-torsional deformations occur during lateral-torsional buckling, it was considered that the narrow inter-branch gaps observed would not be included in the analysis at this stage of the study.

In contrast, the models’ horizontal geometric imperfections after bolting in the 2C built-up section were considered. For this purpose, the lack of straightness was measured in the plane of lower stiffness of the cross-section 2C, between the ends of the free models, i.e., not fixed in the supports. A specially prepared stand was used for the measurements.

**Figure 7:**
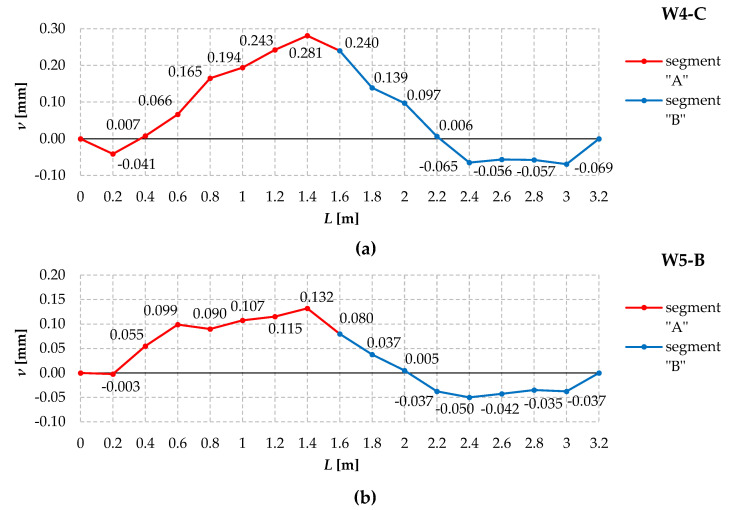
Horizontal geometric imperfections of the (**a**) W4-C model and (**b**) W5-B model.

To measure horizontal geometric imperfections, the 2C test model was divided into two segments, “A” and “B”, separated by support cross-sections and a load application point at the centre of the span (see Figure 1). In this arrangement, the beam was made up of two symmetrical segments, each with a length of 1.6 m. At 20 cm intervals, markers were placed in the segments. A total of 17 markers were marked for the entire 2C model, separating a total of 16 compartments (8 in each segment). Horizontal geometric imperfections were measured using an electronic sensor (24/0.01 mm) mounted on a 3 m precision ruler. The ruler and sensor were moved along the model, measuring the initial lateral deflection relative to the base points every 20 cm. The measuring sensor was positioned so that the sign (direction) of the displacement of its head was in line with the sign (direction) of the displacement of the sensor heads 5, 6, 7, and 8 during the main tests (see Figure 5a). The course of the measured horizontal geometric imperfections for the exemplary models W4-C and W5-B is shown in Figure 7.

It can be seen from the diagrams that the horizontal form of geometric imperfection of the W4-C (Figure 7a) and W5-B (Figure 7b) models forms a single leading half-wave with extremes in the middle of the span. This form in a significant range of the span is “consistent” with the shape of the increments of lateral displacements associated with the first eigenvalue of the critical load (lateral-torsional buckling of beam 2C according to the one half-wave model).

### 2.5. Course of the Study

The primary research was preceded by a trial experiment on a 2C model to test the settings. This allowed for the development of an appropriate research procedure, including (1) the method and sequence of capturing the model in the support bearings, (2) how to apply the ballast load, (3) correcting the sensors settings, and (4) the initial estimation of the critical load.

The test programme for the experimental determination of the critical force *P_z_*_,*cr*_ (cf. Figure 1 and Figure 4) for each of the variants W1 to W-5 (20 2C models in total) included

(1) Setting up the 2C model on the test bench, setting up the loading system (frame–platform), and calibrating the displacement sensors.
(2) Preloading to approximately 10% of the predicted critical force for the variant, checking for the correct model grip, resetting clearances, and checking sensor readings. The sensor readings were reset to zero (step 0) when the model was unloaded.
(3) The 2C model was loaded in successive steps: load platform 26 kg (255 N)—step 1: weights 4 × 20 kg (4 × 196 N)—steps 2 to 5: the sensors’ readings were entered synchronously into a data processing spreadsheet prepared in Excel.
(4) From the sixth loading step onwards, the weight of the accurate weights depended on the current analysis of the behaviour of the 2C member and the recorded load-lateral displacement and load-torsion angle relationship. In successive steps, the weight of the added weights was gradually reduced from 20 kg (196 N) to min. 0.4 kg (3.9 N) in one step. For each load step, readings were taken from the measuring sensors in the same order. In most cases, the addition of weights was terminated at the 28th step of the load.
(5) Measurements were carried out up to a load not exceeding 88 per cent to max. 95% of the expected critical load *P_z_*_,*cr*_. At the end of the study, the 2C model was gradually relieved of weight.

When loading the models with a concentrated force *P_z_* at the centre of their span (Figure 1 and Figure 4), initially, slight increases in lateral displacement *v* (sensors 5–6, 7 and 8) and torsion angle *φ* (5–6) were observed “in the directions” of initial geometric imperfections.

On one side of test model 2C, horizontal sensors 5, 6, 7, and 8, with a pressure of approximately 1.5 N each (see Figure 5a), were placed. This setting was intended to non-invasively “impose” the direction of lateral displacement of the beam in the pre-critical state and, at the same time, protect the sensors from possible damage due to large displacements associated with the loss of model stability. This exceedingly small horizontal forcing (about 6 N) can be interpreted as a specific loading imperfection, which can be recalculated into an equivalent geometric imperfection. According to Eurocode 3-1-1 [6], when calculating the resistance of beams for lateral-torsional buckling according to the second-order analysis, an equivalent bow imperfection can be assumed to occur only in the plane of lower stiffness of the cross-section.

With a gradual increase in load, the form of the lateral deflections and the “direction of increase” of the torsion angle “tended” towards the theoretical first mode of elastic lateral-torsional buckling displacement. Near the critical load *P_z_*_,*cr*_, there was a significant increase in both lateral deflections and the angle of rotation of the 2C cross-section at the centre of the span.

When the models were tested, over the entire load range (*P_z_* to max. 0.95*P_z_*_,*cr*_), no local or distortion loss of stability of the component walls of built-up cross-section 2C was observed.

The normal stress state induced solely by the bending *M_y_* of the models (*σ_max_* = approx. 95 MPa) did not exceed the linear elastic range (i.e., *σ_max_* = 95 MPa < approx. 0.75*R_eL_* = approx. 310 MPa). Note: In the load range up to approx. *P_z_* < 0.7*P_z_*_,*cr*_, there was essentially unidirectional bending (*M_y_*) of built-up cross-section 2C relative to the stronger axis (*y*–*y*). Beyond this load range, bending moments *M_z_* relative to the weaker axis of the cross-section (*z*–*z*) and flexural-torsional bi-moment *B* appeared in addition to *M_y_*. This was due to the increase in the model’s horizontal displacement and torsion angle generated by the progressive lateral-torsional buckling phenomenon. Estimated from the test model’s horizontal displacements and torsion angle, the total stresses from the bending *M_z_* and bi-moment *B* did not exceed approximately 20 MPa. Accordingly, the values of the normal stresses occurring at the maximum stressed point of the built-up cross-section at the centre of the beam span did not exceed the elastic range. No permanent bending of the longitudinal axis of the 2C models examined was observed after unloading.

## 3. Experimental Results

The results of measurements of the displacements and torsion angles of the models (more precisely, the angle of rotation of the 2C cross-section at the centre of the span) as a function of gravitational loading, obtained from experimental tests, made it possible to analyse the behaviour of the built-up member under elastic lateral-torsional buckling conditions. Selected research results are presented below.

Loading the model with a concentrated force *P_z_* at the centre of the span (Figure 1 and Figure 4) induced a linear bending moment distribution in the component segments (“A” and “B”) with a maximum value at the point of load application.

**Figure 8:**
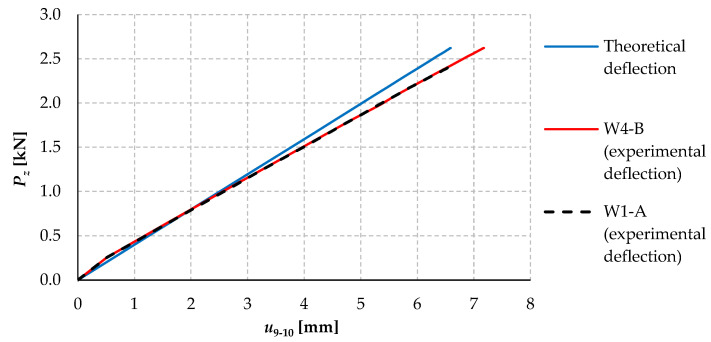
Vertical deflection of beams W1-A and W4-B as a function of load *P_z_*.

Figure 8 compares the plots of the vertical deflection of beams W1-A and W4-B as a function of the force load *P_z_*, with the deflections determined theoretically.

The linear deflection courses of models W1-A and W4-B overlap over the entire load range, as can be seen from the graph (Figure 8). This allows us to conclude that, in the pre-critical loading range (*P_z_* < *P_z_*_,*cr*_), the bolt spacing has no effect on the bending stiffness in the plane of the higher stiffness of cross-section 2C. The experimental deflections were compared with the theoretical deflections (blue line) obtained from the classical analytical formula for the deflection of a beam of a uniform cross-section for the following data: *L* = 320 cm (Figure 1), *E* = 230.825 GPa (Table 3), and *I_y_*_,2*C*_ = 117.76 cm^4^ according to [34].

Further analysis of the graph (Figure 8) revealed that, once the load of approx. *P_z_* = 1.5 kN is exceeded, the increase in deflection determined experimentally is slightly higher compared to the theoretical values. For models W1-A and W4-B, the difference in displacement at a maximum load was approximately 8% (Figure 8). The discrepancy may be due to the method of experimental determination of the vertical deflection, which was determined from the average of the measurements from sensors 9 and 10 (see Figure 5a). As the 2C models also experienced torsional deformation above a certain load level, this may have reduced the accuracy of the vertical deflection readings.

**Figure 9:**
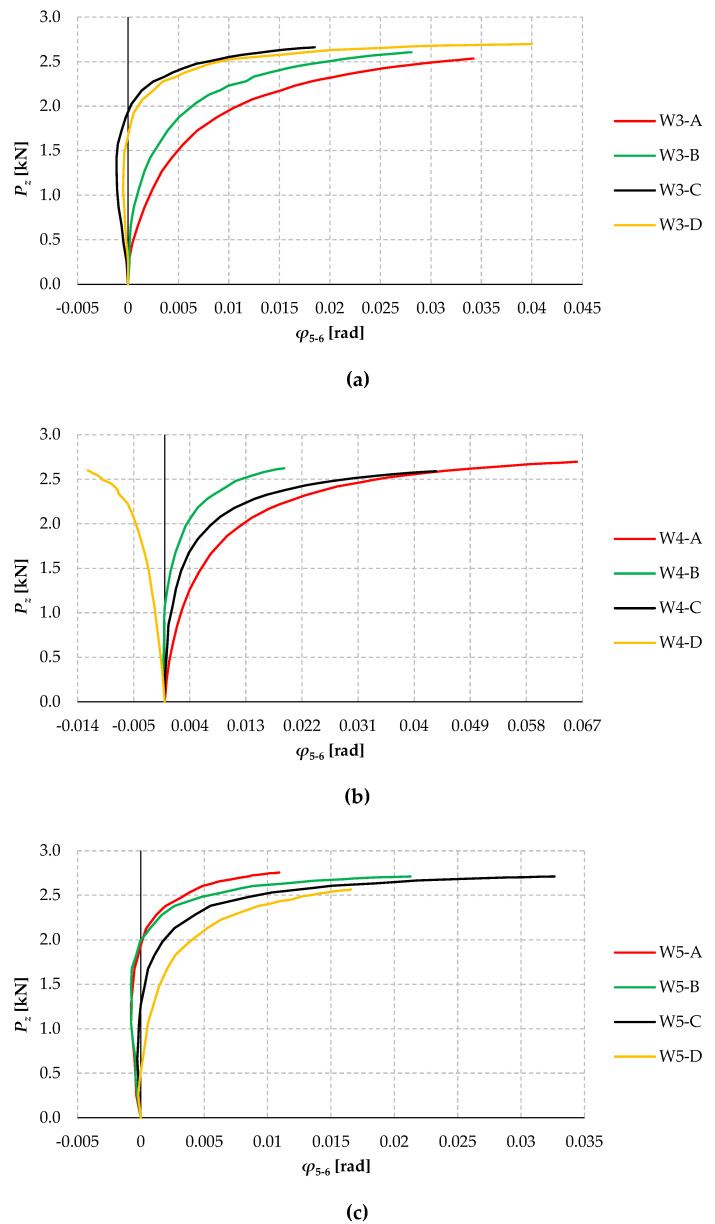
Angles of rotation of the cross-section as a function of load *P_z_* for the (**a**) W3 group, (**b**) W4 groups, and (**c**) W5 groups.

Figure 9 shows the plots of the angle of rotation of the cross-section at the centre of the span of beams W3 (Figure 9a), W4 (Figure 9b), and W5 (Figure 9c) for tests A, B, C, and D as a function of the force load *P_z_*. The angle of rotation was determined in cross-section A-A (see Figure 5a), based on the displacement readings from horizontal sensors 5 and 6, spaced (in vertical direction) on an arm of 140 mm. The effect of vertical deflections on the readings of the rotation angle sensors was compensated by the asymmetric positioning of the sensor heads relative to the gravity centre of the 2C cross-section prior to loading. The position of the sensors was lowered by the value of the expected vertical deflection until the phase of significant horizontal displacement (lowering the sensors by 4 to 5 mm, depending on the model variant). This approach improved the accuracy of the measurements compared to the results from the first trials of the trial model, in which the aforementioned position correction was not applied.

An analysis of the graphs (Figure 9) shows that from a load of approximately 1.0 ÷ 1.2 kN, in most cases, there was a slight non-linear increase in the rotation angle of individual models. In a few cases (e.g., models W3-C, W3-D, W5-A, and W5-B), in the initial stage of loading, the sign of the rotation angle changed. However, in the W4-D model tests, the direction of the beam’s lateral-torsional buckling was opposite to that expected, and the twisted beam exerted pressure on the displacement sensors’ heads. In this case, the slight elastic support of the middle cross-section by the sensor heads (cf. Figure 5) overestimated the value of the critical load obtained (see Section 4.2), and the measurement was finished earlier than for the other models.

At a load of around 2 kN, large non-linear increases in the angle of rotation had already occurred in all cases, indicating that the critical load level was being approached. Subsequent trials within a given group—in this case, W3-A, W3-B, W5-C, and W5-D—were characterised by a greater susceptibility of the models to torsion. This manifested itself in a greater slope and a longer range of static equilibrium paths (*φ*–*P_z_*) at the same load level. This indicates that the influence of the longitudinal geometric imperfections is greater in the models mentioned above. In the range of significant loading (above approx. 2.2 kN), there were already significant non-linear increments in the lateral deflection and rotation angle “in the directions” of the beam dislocations in lateral-torsional buckling.

**Figure 10:**
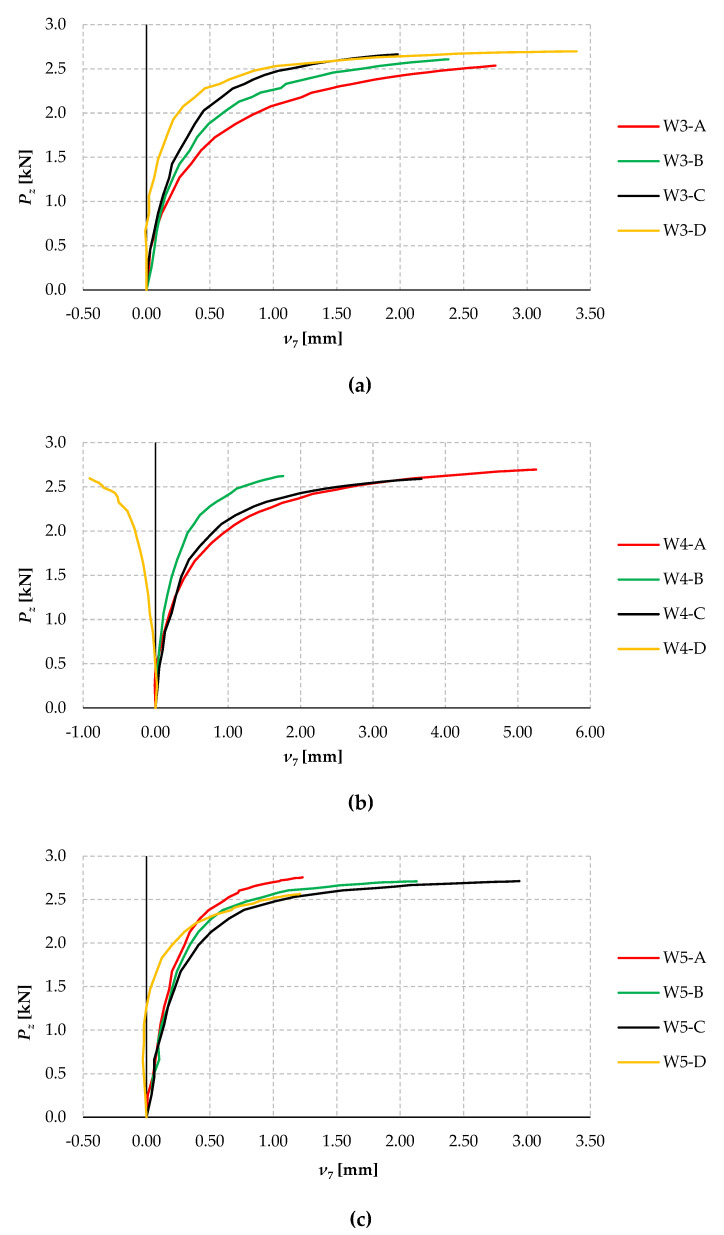
Lateral deflections of beams as a function of load *P_z_* for group (**a**) W3; (**b**) W4; and (**c**) W5.

Figure 10 shows the plots of the lateral displacements of beams W3 (Figure 10a), W4 (Figure 10b), and W5 (Figure 10c) for tests A, B, C, and D as a function of the force load *P_z_*. Lateral deflection was measured with sensor 7 in cross-section C-C (cf. Figure 5a). The effect of vertical deflections of the model on the accuracy of the horizontal displacement reading with sensor 7 was compensated for in the same way as for the rotation angle sensors.

From the analysis of the graphs (Figure 10), it can be seen that from a load of approximately 1.2 ÷ 1.5 kN, non-linear increments in the lateral displacements of the models studied already occurred in most cases. In all cases (except W4-D and W5-D), the horizontal displacements were positive from the initial loading phase (displacement in the direction of the sensor heads). The opposite displacements increment, similar to the increment in the rotation angle (cf. Figure 9b), was found for the W4-D model. Once the load reached approximately 2 kN, in all cases, there was a non-linear and “ordered” increase in displacements in the “direction” of the expected flexural-torsional form of lateral-torsional buckling. It should be noted here that the course of the equilibrium paths (*ν*–*P_z_*) for a given trial (A, B, C, and D) within a group (here W3, W4 and W5) is less differentiated than for the rotation angles (cf. Figure 9 and Figure 10). Compared to horizontal displacements, this indicates a greater variation in the behaviour of the members to torsional deformations.

**Figure 11:**
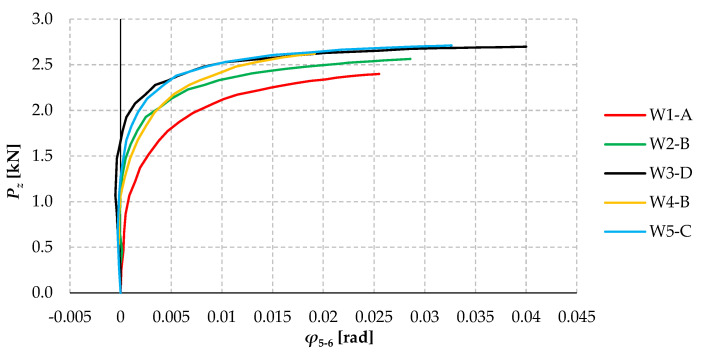
Angles of rotation of selected models from all groups W1 ÷ W5 as a function of load *P_z_*.

Figure 11 shows plots of the rotation angle of selected models from all groups (W1 to W5) as a function of the force load *P_z_*. The rotation angle was determined in cross-section A-A (see Figure 5a) based on displacement readings from the horizontal sensors 5 and 6 (in the same way as described for Figure 9).

Having analysed the graphs (Figure 11), it can be concluded that (1) Model W1 has the lowest torsional stiffness, with only three bolts used to connect the branches, (2) Models W3, W4, and W5 have the highest torsional stiffness, with little difference between them, and (3) Model W2 (5 bolts) has intermediate stiffness.

For models W2 to W5, the non-linear increase in the torsional angle starts in the load range above about 1.5 kN, while model W1 behaves non-linearly from a load of about 1 kN (see Figure 11). In the range of significant loading (above 2 kN), there were significant and non-linear increases in the torsion angles of the models on the “directions” of the flexural-torsional mode of lateral-torsional buckling.

**Figure 12:**
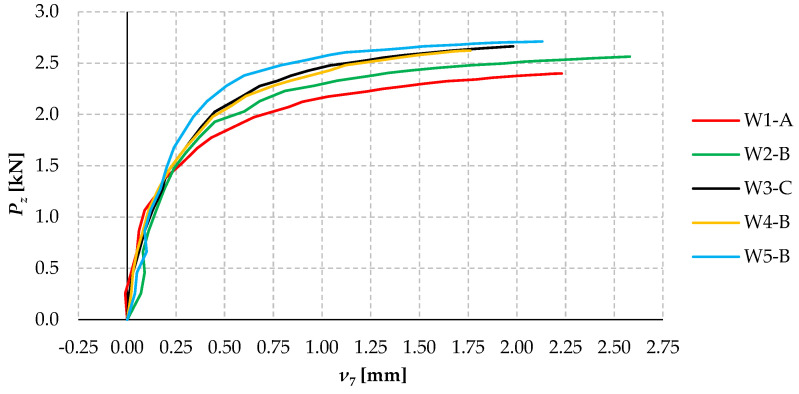
Horizontal displacements of selected models from all groups W1 ÷ W5 as a function of load *P_z_*.

Figure 12 shows plots of the horizontal displacements of selected models from all groups (W1 to W5) as a function of *P_z_* force loading. Lateral deflection was measured with sensor 7 in cross-section C-C (cf. Figure 5a) in the same way as described in Figure 10.

The analysis of the plots (Figure 12) shows that, in most cases, up to a load of approximately 1.5 kN, the horizontal displacements were linear, followed by a slightly non-linear increase. There was a significant and non-linear increase in displacements for all models once a significant load was reached (above 2 kN).

The analysis of the plots indicates that model W1 had the lowest stiffness to lateral displacements caused by lateral-torsional buckling, while model W5 had the highest (cf. Figure 12). This means that the research carried out has demonstrated the influence of the number of bolted connections on the flexural-torsional stiffness of the 2C section. The largest difference in lateral deflection for the aforementioned models occurred at a load of approximately 2.4 kN and amounted to more than 270% (model W1-A: *ν* = 2.23 mm, model W5-B: *ν* = 0.60 mm).

## 4. Elastic Critical Load *P_z_*_,*cr*_

### 4.1. Ordered Deflection Interval

The experimental results presented in Section 3 and observations of the physical phenomena taking place, as well as an analysis of the state of knowledge [28,29,31,32], enabled the authors to generalise the well-known method for the experimental determination of the elastic critical load [25] to the case of the elastic lateral-torsional buckling of built-up members with a 2C cross-section.

In order to determine the elastic critical load *P_z_*_,*cr*_ and the critical moment *M_cr_* (for the static scheme considered) of a 2C beam burdened with random realisations of geometric imperfections, it is necessary to determine the so-called “ordered deflection interval” (ODI). In the ODI, the formed form of deformation (torsion angle, lateral deflection) tends towards the form of lateral-torsional buckling from the condition of the minimum of the total potential energy of the beam–load system.

On the basis of the experimentally determined static equilibrium paths (SEP) *P_z_*–*φ* (lateral force–torsion angle), in the coordinate system (*φ*/*P_z_*, *φ*), “measuring points” plots suitable for ODI determination were drawn up. The expression “measuring point” means the *i*-th pair of coordinates (angle of rotation as a function of load) determined experimentally and represented on the SEP plot as well as the *i*-th pair of coordinates of the same “point” represented on the plot of the so-called reduced system [32].

The method of determining the ODI for the 2C beams analysed in this paper has been simplified compared to the procedure for the experimental determination of *B_cr_* (critical bi-moment) according to the work [32]. This is due to the much lower sensitivity of the beam (with a 2C built-up cross-section of no more than Class 3) to geometric imperfections of the longitudinal axis of the member compared to local imperfections of the component walls of the Class 4 cross-section. The second reason is that the expected subsequent (second and third) eigenvalues of the critical moment (*M_cr_*_,2_—lateral-torsional buckling by two half-waves, *M_cr_*_,3_—three half-waves) are significantly higher than the first eigenvalue (*M_cr_*_,1_—lateral-torsional buckling by one half-wave), which is determined in the experiment. This effect results in significantly less “perturbation” caused by incremental displacements (torsion angle and lateral deflection) on imperfection “directions” associated with higher eigenvalues. The aforementioned phenomenon of the so-called “disturbance of expected displacements”, which occurs particularly in Class 4 sections, is discussed in detail in the work [32].

“Displacement ordering” in this paper means the “fulfilment” by the measurement points, defined in the coordinate system (*φ*/*P_z_*–*φ*), of the so-called displacement amplification equation (here, angles of rotation) according to Equation (1):

(1) $$\varphi_{i}=\frac{\varphi_{0}}{\left(1-\frac{P_{z,i}}{P_{z,cr}}\right)}$$

where $\varphi_{i}$—angle of rotation for the *i*-th measuring point, $P_{z,i}$—load for the *i*-th measuring point, $P_{z,cr}$—elastic critical load, and $\varphi_{0}$—initial angle of rotation of the cross-section at the centre of the beam span 2C.

The method of determining the ODI and estimating the elastic critical load (critical force *P_z_*_,*cr*_) is presented below for the example of the W2-D test model. In this case, the experimental first critical moment *M_cr_*_,1_ of the built-up member 2C loaded with a concentrated force at the centre of the span (cf. Figure 1) can be determined from the classical formula

(2) $$M_{cr}=\frac{P_{z,cr}L}{4}$$

where L—beam span.

**Figure 13:**
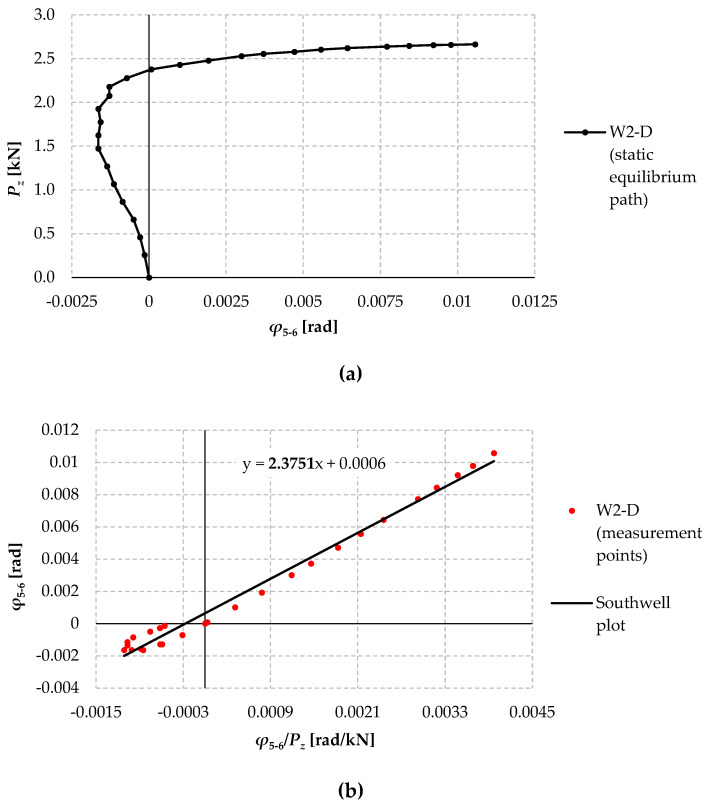
(**a**) Experimental static equilibrium path (*P_z_*–*φ*) of the W2-D model over the entire load range; (**b**) “Measuring points” in the coordinate (*φ*/*P_z_*–*φ*) system.

Figure 13a shows the static equilibrium path: the concentrated force–angle of rotation (*P_z_*–*φ*) determined experimentally for the W2-D model. The rotation angle was measured at the location of the expected extreme displacements for the theoretical first mode of lateral-torsional buckling associated with the first eigenvalue *M_cr_*_,1_. Figure 13b shows a plot of the corresponding “measuring points” in the coordinate system (*φ*/*P_z_*–*φ*).

Carrying out an effective “Southwell straight line regression” for the graph shown below (Figure 13b) is subject to significant error due to the lack of linearity of the designated measured points. In order to effectively determine the elastic critical load (*P_z_*_,*cr*_), a sub-critical ODI range was defined, in which the form of the torsion angle increments of the 2C member with the individual distribution of the initial imperfections tended towards the mode of lateral-torsional buckling from the condition of the minimum of the total potential energy of the system (beam 2C–load).

By analysing the graphs in Figure 13, it is possible to distinguish two main compartments characterising the distinct phases of the static equilibrium path *P_z_*–*φ* of the W2-D model.

Compartment I (points 1 ÷ 10; load up to approximately 2 kN) is characterised by a lack of “order” of the measurement points (see Figure 13b) due to the amplification of the torsion angle on the “directions” of random geometric imperfections. The compartment ends with a kind of “homing” of the rotation angle measurement coordinates to the “correct” path corresponding to the lowest (first) critical load (points 11 ÷ 12).

Compartment II (points 13 ÷ 27, load above 2 kN) is a sub-critical ODI in which there is a continuous increase in the torsional angle on torsional deformation directions caused by a loss of stability and associated with the first critical force *P_z_*_,*cr*,1_ and the first eigenvalue *M_cr_*_,1._ In the coordinate system (*φ*/*P_z_*, *φ*)—Figure 13b, the measurement points of compartment II mark a straight line signalling the range of dominance of the sub-critical rotational angle increments characteristic of the first mode of lateral-torsional buckling.

The method of determining the upper limit of the ODI in compartment II and the assessment of the possible suitability of compartment III (associated with large flexural-torsional deformations of element 2C and preceding the ultimate limit state) for the determination of the elastic critical load will be the subject of further research.

As a basis for the experimental determination of the elastic critical load with the modified Southwell method, measurement points from the sub-critical ODI (compartment II) were considered, in which the torsion angle increments of the 2C element best correspond to the spatial form of the lateral-torsional buckling.

**Figure 14:**
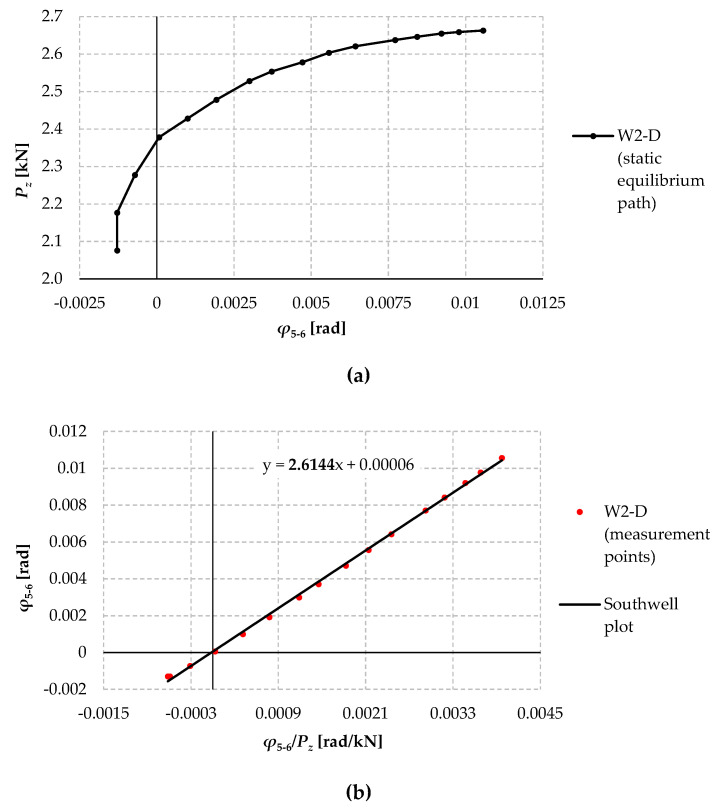
(**a**) Experimental static equilibrium path (*P_z_*–*φ*) of the W2-D model in compartment II; (**b**) “Measuring points” in the coordinate system (*φ*/*P_z_*–*φ*).

Figure 14 shows the computational “measurement points” of compartment II (Figure 14a) and the straight line (linear correlation coefficient above 0.9989) determined on their basis using the least squares method, (Figure 14b). This means that the coordinates of the “measuring points” of compartment II in the coordinate system (Figure 14b) satisfy Equation (1). It was also helpful to analyse the so-called residual errors of the “lower” measuring points of the classified compartment II. This made it possible to discard the coordinates that deviated most from the Southwell straight line and re-designate a straight line for the reduced compartment.

The further procedure is analogous to the classic Southwell method. The directional coefficient of the straight line determines the value of the elastic critical load. In the example under consideration, the directional coefficient of the straight line determines the experimental critical force *P_z_*_,*cr*_ = 2.614 kN from which the elastic critical moment of lateral-torsional buckling *M_cr_*_,1_ = 2.091 kNm can be calculated using formula (2).

### 4.2. Calculation Results

The experimental critical loads of the built-up 2C members for each variant (W1 to W5) were determined according to the procedure described in Section 4.1.

**Figure 15:**
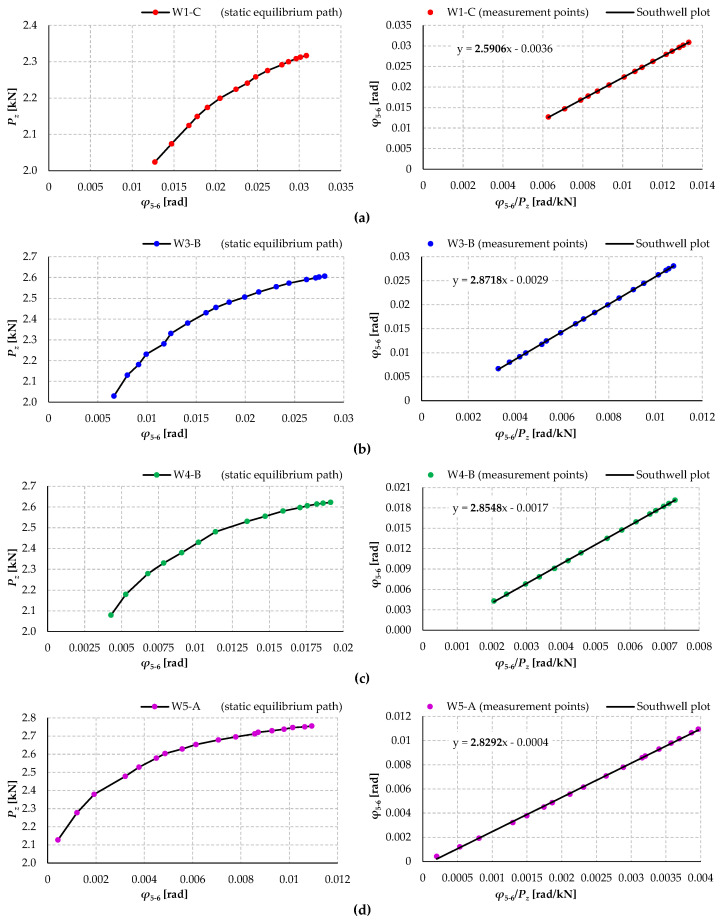
Experimental static equilibrium paths (*P_z_*–*φ*) and Southwell plots for models (**a**) W1-C; (**b**) W3-B; (**c**) W4-B; and (**d**) W5-A.

Figure 15 shows the computational “measurement points” of compartment II and the Southwell plots determined on their basis for selected models of individual groups.

The values of the elastic critical loads obtained on the basis of experimental data for the models studied are shown in Table 4. In column II of Table 4, the designation of the variant of the research model is given, in column III, the designation of the model in the given group (A, B, C, or D) is given, in column IV, the *i*-th critical force ($P_{z,cr,E}$) determined experimentally on the basis of the *i*-th rotation angle diagram in the ODI is given, in column V, the average ($\bar{P_{z,cr,E}}$) value of the critical force within the given variant is given, in column VI, the standard deviation ($s_{x,n-1}$) and the coefficient of variation (V) of the results are given, and in column VII, the relation $\bar{P_{z,cr,E}}/P_{z,cr,N}$ is given, where $P_{z,cr,N}$ denotes the critical force for an analogous uniform I-section determined numerically according to Section 4.3.

A comparison of the values in Table 4 shows that increasing the number of bolts in an individual variant resulted in an increase in the critical load *P_z_*_,*cr*_; however, it was less than expected. Based on the test results (Table 4), it can be concluded that when using bolts from seven upwards, there is no further increase in *P_z_*_,*cr*_. The explanation for this surprising phenomenon will be the subject of further research by the authors of this work.

The coefficients of variation of the elastic critical load obtained in the experiments in individual model variants are insignificant (max. 4.7% for W1) and decrease with an increasing number of bolts (min. 1.3% for W5). This demonstrates the high accuracy of the models and the test bench, the correct way of generating the gravity load, the high accuracy of the rotation angle measurements, and the correctness of the modification of Southwell’s method used in the work to determine the *P_z_*_,*cr*_.

### 4.3. Comparison of Results with FEM Simulations

The elastic critical loads of test models W1 ÷ W5 determined based on experimental measurements were compared with the numerical results obtained with the *LTBeamN* software, Version 2.0.1, [38] (cf. Table 4, Col. VII).

The commonly known *LTBeamN* software, based on thin-walled bar elements (FEM) with seven degrees of freedom at the node, is used to determine the elastic critical moment of lateral-torsional buckling *M_cr_* for a beam with uniform I-sections. Based on *M_cr_*, the elastic critical load of the beam can be calculated.

The FEM calculations considered the beam static scheme adopted in the experimental studies (cf. Figure 1), the method of load application, and the fork support conditions. In the first approximation (first-generation FEM model—thin-walled bar elements), the cross-section of the 2C built-up beam was approximated in the software by a uniform I-section considering the height, width, and thickness of the flanges determined based on the nominal dimensions of the built-up cross-section 2C. The web thickness of the “I” section was defined as 1.26∙*t* (where *t* = 3 mm), which allowed the free torsional stiffness of the two webs of cross-section 2C to be transferred to the single web of section “I”. This procedure allowed for equalising the torsion constant of both cross-sections (“I” and 2C).

For the “I” equivalent cross-section thus defined, the beam was discretised with 100 thin-walled elements, and numerical calculations of the elastic critical moment were performed. Based on *M_cr_*_,*N*_ = 2.671 kNm, the elastic critical load value *P_z_*_,*cr*,*N*_ = 3.339 kN was obtained from Equation (2).

A comparison of the values obtained experimentally (Table 4) with the numerical simulations shows that, in all cases of bolt spacing (variants W1 to W5), the experimental critical loads $\bar{P_{z,cr,E}}$ are lower compared to the values obtained numerically for the uniform cross-section “I”, from 23% for the 3rd bolt (variant W1) to 16% for the 11th bolt (variant W5).

More advanced numerical simulations for the next generation of FEM models (shell elements, solid elements), considering, among other things, contact phenomena, hole clearances, and the degree of pre-stressing of the bolts, will be the subject of further research by the authors.

## 5. Results, Discussion, and Conclusions

Experimental studies have shown that in beams with a 2C built-up cross-section, the phenomenon of lateral-torsional buckling is exhibited during transverse bending. The elastic critical load *P_z_*_,*cr*_ was conditioned by the occurrence of an elastic critical moment of lateral-torsional buckling *M_cr_* with a linear longitudinal distribution (in segments “A” and “B”), with a maximum value in the middle of the span. *M_cr_* induced the compression of the top flange of cross-section 2C and its flexural-torsional deformation with respect to the axis of lower cross-section stiffness. In the experiments carried out, no loss of stability of a single branch between connectors was observed, even for models with three bolts (variant W1). No local or distortion loss of stability phenomena was observed.

For the models loaded with a concentrated force applied to the top flange, a moderate influence of the form of the initial geometric imperfections on the behaviour of the tested beams was found. As the load built up, the initial imperfections (horizontal displacements and torsion angles) were gradually “ordered”, heading towards lateral-torsional buckling according to the first eigenvalue of *M_cr_*_,1_. The observation of the above phenomena allowed the authors to generalise the known method of the experimental determination of a critical load to the elastic lateral-torsional buckling of built-up 2C beams loaded transversely.

The physical form of the spatial loss of stability of the models tested corresponded to the expected form of lateral-torsional buckling for an element with a uniform cross-section (lateral displacement and torsion angle according to one-half-wave with maximum values at the centre of the span). However, lower values of *P_z_*_,*cr*,*E*_ were obtained for the built-up 2C members compared to FEM simulations for the equivalent “I” uniform cross-section.

The general conclusions from the experimental studies are the confirmation of the first three hypotheses:

(1) In transversely bending built-up 2C beams, there is an eigenvalue of the elastic critical load, which was determined experimentally using a modification of the Southwell method. For this purpose, ODIs were designated as presented in Section 4. For the static scheme considered (Figure 1), *P_z_*_,*cr*_ can be converted to *M_cr_* according to Formula (2).
(2) The elastic critical load depends on the longitudinal spacing of the bolt connectors (cf. Table 4). However, the use of more than seven connectors along the length of the built-up member did not increase *P_z_*_,*cr*_.
(3) Loading with ballast on the platform and transferring the concentrated force through the sling frame and ball to the top flange of the test model allowed for only gravity loading without the influence of lateral support. The use of C-section members with cross-sections insensitive to local buckling (a class no higher than third) allowed for the assessment of the behaviour of the built-up 2C member under conditions of elastic lateral-torsional buckling only. The critical moment *M_cr_*, determined experimentally in this way, defines the eigenvalue of the system (beam 2C–load), without the influence of other interaction buckling modes.

However, the experimental investigations carried out did not confirm the thesis that there is a bolt spacing greater than in compression elements and economically justifiable, which provides the critical load capacity as for a uniform element. The maximum compaction of the bolt connectors provided for in this study (*L*_1_ = 2.5 × 15*i_min_*), located at the mid-height of cross-section 2C, did not provide an equivalent critical resistance from the lateral-torsional buckling condition as for the uniform cross-section. The maximum percentage differences are shown in Table 4 (col. VII).

## 6. Concluding Remarks

In transversely loaded built-up 2C beams, the elastic critical load *P_z_*_,*cr*_ can be determined experimentally from the lateral-torsional buckling condition despite the fact that the test models are generally subject to random geometric imperfections. For this purpose, the sub-critical range of the ODI for the angle of rotation of the cross-section at the centre of the span must be determined, in which the longitudinal distribution (function) of the torsion angle of element 2C tends towards a form consistent with the form of lateral-torsional buckling conditioned by the most minor (first) critical moment *M_cr_*_,1_. In this range of a significant load, Equation (1) of the amplification of rotation angles is satisfied.

In the experimental testing of built-up 2C members subject to random geometric imperfections, “searching” for a form compatible with the mode of lateral-torsional buckling is more complex than in compression members and generally occurs at a higher relative pre-buckling load.

Errors in the estimation of critical loads determined by known methods [25,28,29,31,32] based on “measuring points” located below the sub-critical ODI are due to the “perturbation of expected displacements” in the low and medium load ranges “in the directions” of random initial imperfections that deviate from the expected form of stability loss.

However, the correct determination of the ODI allowed for a reasonable estimation of the elastic critical load of transversely bending built-up 2C beams (for a cross-section class no greater than three) regardless of the bolt spacing, provided that their minimum number is three. In this case, the bolts have been placed at the support nodes (one bolt each) and in the centre of the span of the model. At significant lengths of compartments, the possibility of a single branch lateral-torsional buckling between the connectors must be considered, which may occur before the entire built-up 2C member undergoes lateral-torsional buckling. The evaluation of the spacing of the connectors, reducing to a minimum the phenomenon of interaction between the lateral-torsional buckling of a single C-branch and the entire 2C member, will be the subject of further research by the authors.

Further experimental, theoretical, and analytical studies and numerical simulations are recommended to determine the influence of connector spacing on the elastic critical load of built-up members in simple and complex load states. This will allow for the development of adequate computational models for the design of this class of members.

## Figures and Tables

**Table 1:** Designations of test model variants 2C.

No.	Variant Designation		Number of Bolts	Bolt Spacing *L*_1_ [cm]
I	II	III	IV	V
1	W1	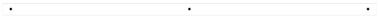	3	160
2	W2	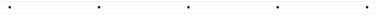	5	80
3	W3	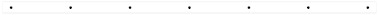	7	53.3 (3)
4	W4	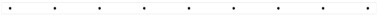	9	40
5	W5	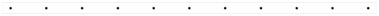	11	32

**Table 2:** Measured dimensions for the w4-4 model.

No.	Dimension Designation	Mean Value $\bar{x}$ [mm]	Standard Deviation $s_{x}$ [mm]	Coefficient of Variation $V_{x}$ [%]	Minimum Dimension [mm]	Maximum Dimension [mm]
I	II	III	IV	V	VII	VIII
1	*b*_1_	29.77	0.1413	0.47	29.60	29.97
2	*b*_2_	100.42	0.1260	0.13	100.29	100.61
3	*b*_3_	29.25	0.3261	1.11	28.90	29.63
4	*t*_1_	2.99	0.0130	0.44	2.97	3.00
5	*t*_3_	3.03	0.0114	0.38	3.02	3.05

**Table 3:** Mechanical characteristics of steel (basic material).

No.	Designation	Young’s Modulus *E* [GPa]	Yield Stress *R_e_* [MPa]		Tensile Strength *R_m_* [MPa]
			*R_eH_*	*R_eL_*	
I	II	III	IV	V	VI
1	Mean value	230.82	422.30	413.19	506.89
2	Standard deviation	3.532	3.485	4.518	4.613
3	Coefficient of variation [%]	1.53	0.83	1.09	0.91

**Table 4:** Comparison of the elastic critical loads of research models W1 ÷ W5.

No.	Variant Designation		$P_{z,cr,E}$ [kN]	$\bar{P_{z,cr,E}}$ [kN]	$s_{x,n-1}$ [kN] (V)	$\bar{P_{z,cr,E}}/P_{z,cr,N}$
I	II	III	IV	V	VI	VII
1	W1	A	2.632	2.571	0.1209 (0.0470)	0.77
2		B	2.395			
3		C	2.591			
4		D	2.665			
5	W2	A	2.829	2.741	0.1124 (0.0410)	0.82
6		B	2.679			
7		C	2.843			
8		D	2.614			
9	W3	A	2.906	2.810	0.0929 (0.0331)	0.84
10		B	2.872			
11		C	2.716			
12		D	2.746			
13	W4	A	2.939	2.855	0.0838 (0.0294)	0.85
14		B	2.855			
15		C	2.771			
16		D	3.112 *			
17	W5	A	2.829	2.803	0.0375 (0.0134)	0.84
18		B	2.756			
19		C	2.789			
20		D	2.836			

* Caution: Because, in the tests of the W4-D model, the direction of the beam’s lateral-torsional buckling was opposite to that expected and the twisted beam exerted pressure on the heads of the displacement sensors, the measurement was finished earlier than for the other models. In this case, the slight elastic support of the middle cross-section by the sensor heads overestimated the value of the obtained critical load. Therefore, the average value of *P_z_*_,*cr*_ for variant W4 was determined based on the results from the three models (A, B, and C).

## Data Availability

The original contributions presented in the study are included in the article, further inquiries can be directed to the corresponding author.
